# Impact of the COVID-19 pandemic on employment and inequalities: a systematic review of international evidence and critical appraisal of statistical methods

**DOI:** 10.1177/17579139241231910

**Published:** 2024-03-13

**Authors:** A Abugamza, D Kaskirbayeva, A Charlwood, S Nikolova, A Martin

**Affiliations:** Academic Unit of Health Economics, Leeds Institute of Health Sciences, University of Leeds, UK; Department of Health Sciences, College of Health and Rehabilitation Sciences, Princess Nourah bint Abdulrahman University, Saudi Arabia; KAZGUU University, Kazakhstan; Leeds University Business School, University of Leeds, UK; Academic Unit of Health Economics, Leeds Institute of Health Sciences, University of Leeds, UK; Real World Methods and Evidence Generation, IQVIA, UK; Academic Unit of Health Economics, Leeds Institute of Health Sciences, University of Leeds, UK

**Keywords:** COVID-19, inequalities, systematic review, work, public health, employment

## Abstract

**Aims:**

To assess the impact of the COVID-19 pandemic on individual labour market outcomes and how these vary over time and between different groups of individuals.

**Methods:**

Searches were conducted using Medline, Scopus and EconLit. Grey literature searches used Google Scholar and Econpapers. Study quality was assessed using the risk of bias in non-randomised studies of exposure tool (ROBINS-E), accompanied by a directed acyclic graph (DAG) to identify relevant mediators, moderators and confounders.

**Results:**

A total of 85 studies (77 peer-reviewed articles, 8 working papers) were included. The ROBINS-E showed that the overall risk of bias varied between studies from low (*n* = 14), moderate (*n* = 56) to serious (*n* = 15). Studies also varied in terms of outcome measures, study designs and the academic disciplines of researchers. Generally, studies using data collected before and during the pandemic showed large negative effects on employment, working hours and income. Studies that assessed moderators (e.g. by industry, occupation, age, gender, race and country of birth) indicated the pandemic has likely worsened pre-existing disparities in health and work. Generally, women, less educated, non-whites and young workers were affected the most, perhaps due to their jobs involving high levels of personal contact (e.g. hospitality, sales and entertainment) and being less amenable to remote working. The DAG highlighted methodological challenges in drawing robust inferences about COVID-19’s impact on employment, including the lack of an unexposed control group.

**Conclusions:**

The COVID-19 health crisis caused unanticipated and unprecedented changes to employment opportunities around the world, with potential long-term health consequences. Further research should investigate the longer-term impact of COVID-19, with greater attention given to low- and middle-income countries. Our study provides guidance on the design and critical appraisal of future studies.

## Introduction

Fulfilling employment and good working conditions are key determinants of physical health, mental health and wellbeing.^1^ On many levels, employment contributes significantly to improving public health. First, having employment gives people a feeling of identity and purpose, which promotes mental health and lowers the risk of mental health problems like depression and anxiety.^2^ In addition, employment is frequently associated with better financial security and expanded access to healthcare services, allowing people to get timely medical care and adopt healthier lives.^3^ Work also provides opportunities for learning, social interaction, nurturing personal identity and self-esteem. Supporting people to remain in work is therefore an important goal of public health policy.^4^

The COVID-19 pandemic caused unanticipated and unprecedented changes to individual employment work conditions and labour markets around the world. This was partly due to people adjusting their purchasing and labour supply decisions because of ill health from COVID-19 infection, economic uncertainty or to avoid catching COVID-19; and partly due to restrictions on economic activity imposed by Governments to reduce the spread of COVID-19. In an immediate response to the COVID-19 outbreak, unemployment rose steeply from 4% to 5% to slightly more than 11% in Australia^5^, and more than 14% in the US.^6^ In contrast, unemployment rates rose less dramatically in Europe where comprehensive furlough schemes were introduced. The pandemic negatively affected almost all sectors, but some industries like travel and hospitality were hit harder than others due to reduced consumer demand for their goods or services.^7^ More recently, economies are emerging into a ‘new normal’, with some people enjoying new opportunities while others face ongoing challenges.

Despite an increasing number of publications on COVID-19’s public health impact, there is a lack of synthesised evidence on the impact of the pandemic on various individual-level labour market outcomes (LMOs), such as job loss, employment and changes in wages or working hours, and how this varies between different individuals. A recently published narrative review^8^ suggested there was no major impact of COVID-19 on employment status, working hours and earnings after controlling for publication bias (i.e. bias that occurs when the publication of a study depends not just on the quality of the research but also on the significance of the results). However, the review was limited to 29 studies conducted prior to February 2021 (25 of which were not peer-reviewed) and it relied on undocumented search strategies implemented in Google Scholar. No consideration was given to heterogeneity of the impact by different background characteristics.

Therefore, this review aimed to comprehensively synthesise available evidence regarding the impact of the COVID-19 pandemic on individual-level LMOs. The objectives were as follows:

1. To critically assess the quality of existing evidence regarding the impact of the COVID-19 pandemic on various dimensions of individual LMOs, such as employment status, income levels and working hours.
2. To explore variations in the impact of the pandemic on LMOs among different demographic groups depending on gender, age, ethnicity or job type and explore implications for inequalities in LMOs.
3. To contribute to the methodological aspects of public health research by providing insights into the critical appraisal of studies examining the impact of the COVID-19 pandemic on individual-level LMOs and inform the design of future research in this domain.

A particular feature of research on this topic is that it comes from many different disciplines that do not always use the terminology and statistical methods that are widely recognised in public health research. Another feature is that due to a need for COVID-19 data collection and analysis to be conducted rapidly, many studies used novel observational datasets and were published using an expediated peer-review process. Interpretating and judging the quality of these studies is therefore important, but potentially challenging. To address this, our systematic review utilises contemporary techniques from the fields of medicine and epidemiology for the synthesis and quality appraisal of observational studies, including the risk of bias (RoB) in non-randomised studies – of exposure (ROBINS-E) tool^9^ and a directed acyclic graph (DAG).^10^ The ROBINS-E tool enabled us to provide a comprehensive assessment of existing studies against the same rigorous criteria, while also making recommendations for the design and critical appraisal of future studies. The DAG is developed a priori and provides a visual representation of causal relationships of the variables beyond the exposure and outcome of interest, including moderators (variables that may modify the impact of COVID-19 on LMOs such as job type) and mediators (variables that represent an effect of COVID-19 and influence its impact on the LMOs such as catching COVID-19 infection). It was incorporated as part of the assessment process, aligning with the first step of the ROBINS-E tool, where reviewers are required to prespecify relevant confounders.

## Methods

The protocol for this review was registered with the Open Science Framework (OSF).^11^ The review reporting process is based on the Preferred Reporting Items for Systematic Reviews and Meta-Analyses (PRISMA) guidelines.^12^

### Search strategy

A.A. explored and tested a variety of search strategies which were finalised in discussions with A.C., A.M., D.K. and S.N. A.A. discussed the search strategy with an information specialist (J.W.) before the full database search. This was done to ensure transparency of the strategy and that it covers the inclusion criteria of the systematic review. The final search strategy included terms and keywords for ‘COVID-19’ and ‘labour market outcomes’, combined by the AND and OR functions as shown in Table 1. Due to the differences in MeSH terms, wildcards and Boolean operators, the search strategy was modified for each included database (see Supplementary Material Appendix 1).

**Table 1.** A simplified search strategy.

Search term	Search strategy
1. COVID-19	“coronavirus” OR “coronavirus*” OR “corona virus” OR “covid” OR “covid19” OR “covid-19” OR “covid 19” OR “2019 ncov” OR “2019-ncov” OR “ncov19” OR “ncov-19” OR “2019-novel” OR “sars-cov-2” OR “CoV” OR “nCoV” OR “Pandemi*”
2. Labour market outcomes	“work” OR “employ*” OR “job” OR “labo?r market” OR “unemployment” OR “job loss” OR “work*hours” OR “retirement” OR “salaries” OR “income” OR “earnings” OR “wages”
3. Boolean logic	(1) AND (2)

Three electronic databases were searched with the predefined search strategy: Medline, Scopus and EconLit. These databases complement each other by covering the distinct research fields related to our review. Forward search was conducted using Google Scholar to explore studies that already cited a given paper. In addition, manual identification of relevant papers (including grey literature and preprints) was performed by A.M., A.C. and A.A. using websites such as Google Scholar and Econpapers.

The full search was conducted by A.A. and included papers published up to 31 January 2022. The results from the three databases were combined using Endnote.^13^ Two reviewers independently filtered studies in Rayyan.^14^

### Eligibility criteria

The Population, Exposure, Comparator and Outcomes (PECO) framework^15^ was used to align the selection process with the review objectives. Table 2 presents the PECO framework developed for this review.

**Table 2.** PECO framework.

P (population)	People were employed pre COVID-19 pandemic (aged > 16 years)
E (exposure)	COVID-19 pandemic
C (comparator)	Comparison of individual-level LMOs between pre COVID-19 and during COVID-19
O (outcome)	Individual-level LMOs during COVID-19, including employment and/or unemployment status, job loss, income and working hours

LMOs: labour market outcomes.

Studies were excluded if they were:

- Published before 2020 (to avoid studies of other types of coronaviruses, e.g. Severe Acute Respiratory Syndrome (SARS));
- If the perspective of the study was the employer (or business) rather than the employee;
- Reporting impact of COVID-19 on non-LMOs (e.g. job search, food consumption, poverty, charity and insurance);
- Non-English.

### Study selection

The identified studies were assessed independently by A.A. as well as D.K. and A.M. using the prespecified inclusion and exclusion criteria in a two-step process. First, titles and abstracts were screened. Second, full texts of those articles that were considered to be eligible were retrieved for further assessment of eligibility; 20% of independent cross-checking was done by the reviewers to minimise selection bias.

### Data extraction

Data were extracted independently by three reviewers (A.A., D.K. and A.M.) using a data extraction tool developed for this systematic review.

### Quality assessment

**Figure 1.**
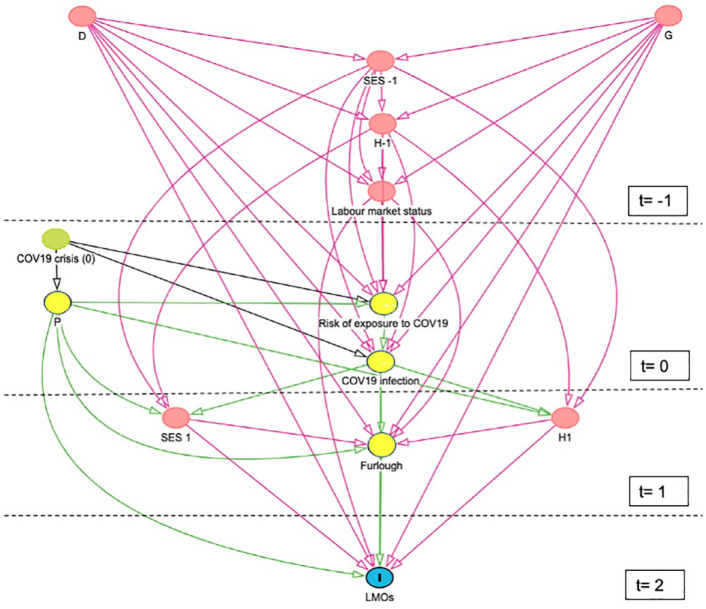
DAG representing the theoretical framework of the COVID-19 impact on labour market outcomes. The numbers represent time dimensions before (*t* = −1) and during (*t* = 1 and *t* = 2) the COVID-19 crisis (which occurred in March 2020 at *t* = 0). Colour code – red: moderator; green: exogenous shock/exposure; yellow: mediators (risk of exposure, infection, furlough); blue: outcome. D: demographic characteristics (age, sex, ethnicity); G: geographical characteristics (country, region and population size); SES: other socioeconomic status (education, marital status and number of children at home); H: health status (physical and mental health); COV19: the coronavirus (COVID-19) pandemic; P: policy interventions (country- or region-specific travel regulations, lockdowns, shielding, stores closures, school closures); LMOs: labour market outcomes.

Three reviewers (A.A., D.K. and A.M.) assessed RoB by using the ROBINS-E risk of bias tool (see Supplementary Material – Appendix 2). Prior to formally assessing the RoB in each study, the tool requires reviewers to describe the context of the review question. This was done using a DAG (Figure 1), which was designed for this review based on pre-existing theory and evidence from previous labour market shocks. It also requires reviewers to provide a hypothetical description of an ideal randomised controlled trial (RCT). Although an ideal RCT would have people randomly allocated to the COVID-19 pandemic, in reality COVID-19 impacted the whole population and, as such, there is no control group that was not exposed.

To develop the DAG, we followed a systematic approach using the DAGitty package.^16^ Our approach was guided by existing research on the COVID-19 pandemic and emerging trends reported in the media at the time. This allowed us to graphically demonstrate both the causal relationships and the logical temporal order of events in our context, as illustrated in Figure 1, with events moving downward from the top:

- *t*-1 represents the time prepandemic and includes individual characteristics which may be moderators of the relationship between COVID-19 and changes in LMOs (i.e. demographic and geographical characteristics, socioeconomic and health status) as well as prepandemic labour market status;
- *t* = 0 represents the onset of the pandemic. Since this was an exogenous exposure affecting all of society, there are no arrows directed towards COVID-19 and there are no potential confounding variables. However, three variables are expected to influence the impact of the pandemic on LMOs. These mediators include the risk of catching the infection, having ill health due to infection and country- or regional-level policy interventions (e.g. lockdowns);
- *t* = 1 represents the time after lockdown started and includes eligibility for furlough (and other mediators such as ability to work from home) and changes to health and socioeconomic status (moderators);
- *t* = 2 is the time period in which changes in LMOs due to COVID-19 are observed (e.g. reduced income or job loss following lockdown).

The DAG implied that background demographic characteristics (age, sex, ethnicity), geographical characteristics (e.g. country, region and population size), job characteristics (job type, job sector, income and working hours), other socioeconomic factors (education, marital status and number of children at home) and health status (physical and mental health) are potential moderators of the COVID-19 impact on LMOs. Therefore, these variables should be considered in building statistical models aimed to measure the COVID-19 impact on LMOs.

### Narrative synthesis

We performed a comprehensive narrative synthesis of the collected data from included studies. This qualitative synthesis describes the patterns and trends regarding how the COVID-19 pandemic has affected individual LMOs in terms employment status, income, working hours and variation between groups of individuals. Our synthesis of the included studies involved a review and interpretation of the results from each study.

Our analytical approach integrated a comprehensive quality assessment of RoBs (section ‘Quality assessment’) with a narrative synthesis to offer a comprehensive evaluation of the effects induced by the COVID-19 pandemic on LMOs.

## Results

**Figure 2.**
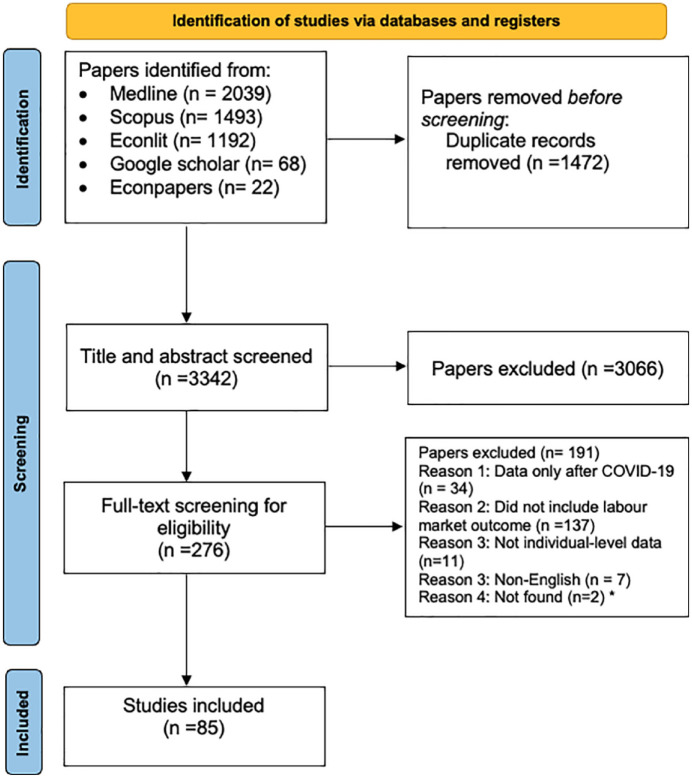
PRISMA flow chart of the included studies. *Studies were requested by email, with no response.

The database searches yielded an initial list of 4814 records. After filtering titles, abstracts and full texts, 85 studies (77 peer reviewed articles, 8 working papers) met our inclusion criteria and were included for data extraction. The PRISMA flow chart in Figure 2 illustrates the identification, screening and inclusion of the studies for this review.

### Study characteristics

Tables A1 and A2 in Supplementary Material Appendix 3 present a summary of the study characteristics and main results of the included studies. The majority of studies (80%) investigated LMOs in high/middle income countries (HMICs) such as Canada, the US, the UK and Australia. The main data source was secondary data from routinely collected surveys (71%). The most repeated data sources were the Current Population Survey (CPS) in the US (*n* = 16), national Labour Force Surveys in Australia, Japan and Europe (*n* = 14) and the ‘Understanding Society’ (UKHLS) household panel survey in the UK (*n* = 5). Most of the included studies (82%) assessed a short-term impact during the first 4–6 months of the pandemic (i.e. March–August 2020). The studies were categorised based on study design as studies that used regression analysis (Table A1) including quasi-experimental methods (e.g. difference-in-differences, interrupted time-series) or descriptive studies (Table A2) that reported descriptive statistics (e.g. rates, percentages, prevalence).

### Quality assessment results

The included studies were judged using the ROBINS-E tool to be at either ‘low’ (16.5%), ‘moderate’ (65.8%) or ‘serious’ RoB (17.7%) as shown in the last column of Tables A1 and A2. Most of the studies that used regression analysis (66.7%) were at moderate RoB, few studies (12.5%) scored low RoB, while 20.8% were at serious RoB due to selection, missing data or both. The descriptive studies showed a higher percentage of low RoB (21.6%), lower percentage of moderate RoB (64.8%) and only 13.6% of the descriptive studies scored serious RoB mainly due to selection. None of the experimental or descriptive studies scored ‘critical’ RoB.

Generally, most of the included studies were judged as having a moderate risk of selection bias (69%), classification bias (72%), measurement of outcomes and selection of the reported results (82%). On the contrary, few studies (14.5%) were at serious risk of selection bias. One reason for being at risk of selection bias was excluding self-employed people who represent an important part of the labour force due to difficulty in calculating their net monthly income. Most studies (66%) did not report information about missing data; however, two studies did report appropriate methods to handle missing data (e.g. multiple imputation) and were thus judged as low RoB in this domain. There was no RoB in any study due to confounding or departures from intended exposures since, as indicated in our DAG, all study participants were impacted by COVID-19.

### Narrative synthesis of LMOs

The studies reported disruption in LMOs across the world following the COVID-19 pandemic. The impact on LMOs is shown in columns 6–8 in Tables A1 and A2 among different types of LMOs. The HMICs studies reported decreases in employment rates by 14%–15% in Canada (*n* = 2 studies), 11%–13% in the US (*n* = 3 studies) and 4.3% in the UK (*n* = 1 study). The unemployment rate increased by 1.1% in Australia and Germany, and 1.40% Spain (*n* = 1 study each). The few studies (14%) from low/middle-income countries (LMICs) showed a higher percentage of job loss, for example, 25% in Senegal, Mali and Burkina Faso,^17^ and 31% in India.^18,19^ In Malaysia every 1% increase in the lockdown measures resulted in almost 1% job loss.^20^ Overall, about half of identified studies were reporting the effects of the pandemic in a broadly descriptive sense. Since these studies involved before and after comparisons, none claimed a causal impact of COVID-19 on LMOs.

Two-thirds of studies examined the moderating effect of ethnicity, gender, age, education or job type on the relationship between COVID-19 and LMOs. For example, four studies reported that Black, Hispanic and Asian employees showed worse economic effects compared with their White counterparts. The US study^21^ reported the largest ethnic inequality percentages, black men were 73% more likely to lose their job than white men, Hispanic men were 95.2% more likely to lose their job than white men and Asian men were 93.7% more likely to lose their job than white men.

Globally, women faced worse employment outcomes, including a larger reduction in working hours and earnings compared with men. One study^22^ reported that women were 24% more likely to lose their job and expected their labour income to fall by 50% more than men. In terms of working hours, mothers with young children have reduced their work hours 4 to 5 times more than fathers.^23^

In terms of education and age, less educated and young workers were more likely to work in occupations most difficult to be done remotely,^24^ to be furloughed^25^ and to experience decline in income.^26^

Several studies (14.5%) reported large negative effects of the pandemic on certain industries and job types (e.g. healthcare, self-employment). For example, Lemieux et al.^27^ reported that in Canada, the impact was larger for self-employed, private sector employees and workers in public-facing occupations such as sales compared with the flexible/low-contact occupations. Another study by Winkelmann and Games^28^ reported a larger impact among professions with minimal computer use such as athletic trainers. Their results showed that about 20% of the included athletic trainers were unable to work at all during lockdown, and most of the employed athletic trainers faced job status changes of either reduction in pay or working hours. In addition, a study from South Korea^29^ reported significant job losses (almost 1 million jobs) in the service sector. Wholesale, retail, food and lodging businesses or educational services were the most affected.

Tsurugano et al.^30^ highlighted the COVID-19 impact on part-time and low-skilled workers such as working students. Their results showed a significant reduction in the number of working students (around 50%) in April 2020 compared with the same period in 2019. Also, disabled workers were more likely to use the government support scheme or be temporarily away from work as reported by Jones.^31^

Furthermore, two studies examined the COVID-19 effects on immigrant workers.^32,33^ The results showed a decline in employment and earnings for immigrants compared with natives. The authors claimed that the reason behind this negative impact is that immigrants are less likely to work in jobs that could be performed remotely.

Two studies reported findings that might have been unanticipated in terms of job type effects. Bhandari et al.^34^ reported a significant increase in unemployment rates among healthcare workers in the US. This included higher unemployment rates among dentists (41.3%), technologists/technicians (10.5%) and home health providers (7.8%) compared with nurses (4%), surgeons (1.4%) and pharmacists (0.7%). Gomes et al.^35^ reported similar findings in terms of reduction in income and workload of Brazilian urologists.

We also found that only one study investigated the impact of catching COVID-19 infection on LMOs,^36^ a key mediating variable. This might be due to the unavailability of testing data, especially in the early stage of the pandemic. The results showed that 30.5% of patients reported a deduction in their salary during the illness and 3.2% reported job loss.

### Statistical methods

The analytical approaches used in the included studies are shown in Table 3. Most reported aggregated descriptive comparisons of LMOs during COVID-19 to data of the same months in a prior year (50%). Some of these studies (16%) used statistical tests such as ANOVA, *t*-test, Chi-square or Fisher’s exact test.

**Table 3.** Summary of LMO measures.

Category	Measurement	Descriptive studies methods		Regression analysis methods
		Descriptive statistics	Statistical tests	Regression models
Unemployment/employment	Unemployment rate	Simple pre/post COVID-19 analysis at national level Calculating the number of unemployed persons as a proportion of the total labour force (unemployed and employed) Prevalence of each type of job transition in the overall sample and by sociodemographic groups and occupation Month-to-month change in absolute volume of employment and labour market entry/exit. Monthly trend analysis of key employment outcomes	Chi-squared test ANOVA test *t*-test	Multivariable linear regressions Person-fixed-effects models Couple-level fixed-effects models Intersectional analysis Fourier causality test
	Job loss	Descriptive statistics for the relevant outcomes aggregated across countries (e.g., rates of job loss and business closure)	Two-tailed two-sample tests Cross-tabulations	Fixed-effects regression models
Income	Wage	Calculating the wage loss as the proportion of the annual wage no longer received given inability work due to COVID-19	Chi-squared or Fisher’s exact test	Standard probit model Interrupted time-series Difference-in-differences
	Salary	Track the impact of government policies (COVID-19 lockdown) on monthly income using data from bank records	N\R	N\R
	Household income	N\R	Pearson’s chi-square test	Household-level fixed-effects regressions Path analysis model Difference-in-differences
Working hours	Working from home	WFH feasibility index Descriptive statistics to provide an overview of general patterns of work	N\R	N\R
	Working at office	Percent change in aggregate weekly hours worked. Subtracting usual hours from actual hours. A positive (negative) difference between these two measures indicates that a particular individual is working more (less) than usual	Chi-squared or Fisher’s exact test	Multivariable linear regressions Person-level fixed effects models Difference-in-differences

ANOVA: one-way analysis of variance;WFH: working from home.

The remaining studies reported fitting multivariate regression models which controlled for demographic and socioeconomic characteristics. Of these studies, some authors reported using specific statistical approaches such as difference-in-differences (9%), interrupted time-series and Fourier’s causality test (3%), which offer potential for more robust causal inference, for example, by providing a counterfactual comparison group. However, none of the reviewed studies included or mentioned using DAGs or other systematic approaches to selecting covariates.

## Discussion

The review highlights the large impact of the COVID-19 pandemic on labour markets across the world. Compared with HMICs, LMICs experienced a higher percentage of job loss due to COVID-19. However, compared with HMICs, the research on LMICs was limited, perhaps due to lack of data in the early stage of the pandemic or limited possibility of field surveys. Another possible reason is the lack of political interest or funding for research on COVID-19’s longer term economic impact, with more attention and support to research on the more immediate COVID-19 health outcomes.

Our results showed variation between the included studies in terms of the outcome measures, study designs and the overall RoB, which precluded a meta-analysis. Overall, all the studies suggested that COVID-19 had a negative effect on employment, income and working hours. The studies of moderators (e.g. by industry, occupation, age, gender, race and country of birth) indicated that the COVID-19 pandemic has likely worsened pre-existing gaps in LMOs. Generally, women, less educated, non-whites and young workers were most affected, perhaps due to their jobs involving high levels of personal contact (e.g. hospitality, sales and entertainment) and being less likely to work in occupations that can be done remotely.^24^ The significant negative impact on women LMOs was linked to their informal care responsibilities – not only to preschool and school-aged children, but in some cases care for older parents with health conditions and relatively more risk to COVID-19.^37^

It is difficult to draw robust causal inferences about the COVID-19 impact on LMOs. About half of the included studies are broadly descriptive with limited methodological strategies. This might be due to limited quality data, research support or slow administrative processes (e.g. ethical approvals) during the early stages of COVID-19. Another likely explanation as shown in the DAG is that COVID-19 represents an exogenous shock to global LMOs, making it difficult to obtain a suitable control or counterfactual group. Also, there was limited consideration of time-varying characteristics of workers that may have changed due to COVID-19 lockdown, some of which would affect causal inference but may be unobserved, such as mental health. One viable strategy is therefore to use interrupted time-series (ITS) models by using preintervention trends to determine what the LMOs would have been in the absence of the COVID-19 pandemic. However, ITS require high-quality pre COVID-19 secondary data collected at multiple time points, which is unlikely to exist in all settings (e.g. LMICs). One of the included studies reported using ITS design,^38^ however, the authors provided little detail about their model design. Although many identified studies had used regression analysis, none reported using a systematic approach to selecting covariates (e.g. DAG). Given that we highlight that there are no potential confounding variables in this setting, this meant that it was not always clear why those covariates had been used.

To our knowledge, this is the first systematic review of COVID-19 impact on LMOs. Demena et al.^8^ conducted a non-systematic narrative review of the short-term impact of the COVID-19 pandemic on LMOs using meta-analysis techniques, with a focus on detecting publication bias in the included sample. No consideration was given to the heterogeneity of the impact by different background characteristics (e.g. age, gender, race and job type), which has been addressed in this review.

Our review is unique in its focus on exploring and assessing the methods used to investigate the COVID-19 pandemic effects on several LMOs. For example, we found that half of the studies used descriptive statistics, which implies the need for further research to statistically investigate the causal effects of COVID-19 on LMOs. Also, this review uniquely captures the moderators, mediators and confounders in the COVID-19 economic context using a theoretical diagram (DAG). The DAG helped us to communicate our understanding of the potential moderators and mediating variables that may influence the pandemic impact on LMOs.

A major strength of our review is the systematically synthesised evidence and use of manual search which helped to pick up studies not available in Medline and Scopus (e.g. working papers). Furthermore, this is the first review that utilised DAG to supplement critical appraisal tool which can inform the critical appraisal and design of future studies.

However, this review has some limitations. First, we excluded studies published in languages other than English, which could mean some countries were overlooked in this review. It is also possible that due to lack of funds, some LMICs did not publish their work and therefore we could not include them. As a result, our conclusions are based on the available evidence that satisfied our criteria.

In addition, it is important to note that while the ROBINS-E tool is widely employed in epidemiology to evaluate RoB in observational research, there are limitations associated with its application. First, two of the six domains of the tool (‘confounding bias’ and ‘departures from intended exposures’) are not applicable to COVID-19 research due to all study participants being affected by COVID-19, as we identified in our DAG. Second, the existing literature highlighted that this tool can be laborious and time-consuming to implement.^9^ Despite the limitations of this tool, it was the best fit for critical appraisal of the included studies and indicates the need for further development of critical appraisal tools for observational studies.

Furthermore, the DAG, although useful in visually representing causal relationships among variables, is not without its own limitations. It may pose challenges for individuals from different disciplines who are less familiar with DAGs, making navigation difficult. Moreover, it becomes unwieldy when numerous variables are added, especially in contexts involving multiple time points.

Looking ahead to future research, most of the existing evidence measured the impact of COVID-19 on LMOs in HMICs. Thus, there is a pressing need for future investigations in LMICs, not least to assess between country differences in COVID-19 impact. In addition, an important area for further research is the longer-term economic impact of COVID-19. This could include raising rates of economic inactivity some years after the COVID-19 restrictions ended, and the impacts of long-Covid on individual LMOs. Building on our work, the developed DAG can be used in similar studies.

## Conclusion

This review documented the unequal impact of COVID-19 depending on individual and job characteristics, which likely led to further widening of pre-existing inequalities in LMOs and health. Evidence from previous systematic reviews showed that moving into unemployment from being at work can be harmful for both physical and mental health. Therefore, it is vital that further rigorous research is conducted on COVID-19, including the longer-term effects. Such research can inform policies, including by supporting the design and targeting of employment and income support interventions, to promote the health and labour outcomes of workers.

## Supplemental Material

sj-docx-1-rsh-10.1177_17579139241231910 – Supplemental material for Impact of the COVID-19 pandemic on employment and inequalities: a systematic review of international evidence and critical appraisal of statistical methods Supplemental material, sj-docx-1-rsh-10.1177_17579139241231910 for Impact of the COVID-19 pandemic on employment and inequalities: a systematic review of international evidence and critical appraisal of statistical methods by A Abugamza, D Kaskirbayeva, A Charlwood, S Nikolova and A Martin in Perspectives in Public Health
