# Supplementary material for: Impact of the COVID-19 pandemic on employment and inequalities: a systematic review of international evidence and critical appraisal of statistical methods
Source: Perspect Public Health. 2024 Mar 13;146(2):85–94. doi: 10.1177/17579139241231910 (PMC13091924; doi:10.1177/17579139241231910)
Supplement: sj-docx-1-rsh-10.1177_17579139241231910 – Supplemental material for Impact of the COVID-19 pandemic on employment and inequalities: a systematic review of international evidence and critical appraisal of statistical methods [file sj-docx-1-rsh-10.1177_17579139241231910.docx]

**Supplementary material Appendix 1: Search terms**

EconLit, Medline and Scopus were searched for studies published after December 2019 that contained any of the following terms in their title, abstract, keywords or topic:

EconLit via EBSCOhost:

The search string: (TX coronavirus or covid-19 or 2019-ncov or sars-cov-2 or covid19) AND (TX work OR employ* OR job OR "labo#r market*" OR unemployment OR "job loss" OR "work* hours" OR salaries OR income OR earnings OR wages OR retirement).

Medline via OVID

Medline expert search for covid19: (2019-ncov or ncov19 or ncov-19 or 2019-novel CoV or sars-cov2 or sars-cov-2 or sarscov2 or sarscov-2 or Sars-coronavirus2 or Sars-coronavirus-2 or SARS-like coronavirus* or coronavirus-19 or covid19 or covid-19 or covid 2019 or ((novel or new or nouveau) adj2 (CoV or nCoV or covid or coronavirus* or corona virus or Pandemi*2)) or ((covid or covid19 or covid-19) and pandemic*2) or (coronavirus* and pneumonia)).ti,kw. limit 1 to yr="2020 -Current" AND (work or employ* or job or "labo?r market*" or "job loss" or "work* hours" or salaries or earnings or wages or retirement).ti,kw.limit 5 to yr="2020 -Current" OR Mesh headings: (employment/ or unemployment/ or income/ or occupations/) limit 3 to yr="2020 -Current"

Scopus Via Elsevier

( TITLE-ABS-KEY ( coronavirus  OR  covid-19  OR  2019-ncov  OR  sars-cov-2  OR  covid19 )  AND  TITLE-ABS-KEY ( work  OR  employ*  OR  job  OR  "labo?r market"  OR  unemployment  OR  "job loss"  OR  "work*hours"  OR  retirement  OR  salaries  OR  income  OR  earnings  OR  wages ) )  AND  PUBYEAR  >  2019

**Supplementary material Appendix 2: Application of the ROBINS-E:**

**Step 1: Scoping review question and identifying confounders.**

AA developed a DAG which was discussed and agreed with AC, AM and SN. The DAG is a non-parametric tool used to represent the relationships between a set of variables and the assumed data-generating process of a specific context [14]. It (**Figure.1**) was generated using DAGitty browser [15]. The design is based on pre-existing theory and evidence from previous labour market shocks. The aim of the DAG was to identify the key variables in relation to our research question.

**Step 2: Hypothetical description of an ideal randomized controlled trial**

As COVID19 impacted everyone, we cannot observe the counterfactual of no COVID-19 exposure.

**Step 3: RoB Assessment**

Using the ROBINS-E template, AA created a custom RoB template in Microsoft Excel which included the seven RoB domains and signalling questions relevant to our review topic. For each study, the ROBINS-E tool (supported in domain 1 by the DAG) was used to evaluate the RoB on each of the seven domains as ‘low’, ‘moderate’, ‘serious’, or ‘critical’.

The overall risk of bias in each study was then judged as follows:

- “Low” RoB: if all RoB domains were classified as ‘low’ RoB.
- “Moderate” RoB if one or more domains were classified as moderate but none as serious or critical.
- “Serious” RoB: if at least one domain was classified as serious but none as critical.
- “Critical” RoB: if at least one domain was classified as critical.

## **Supplementary material Appendix 3: tables A1 and A2.**

| Table A1 Summary of regression analysis studies’ characteristics | | | | | | | | | | |
| --- | --- | --- | --- | --- | --- | --- | --- | --- | --- | --- |
| First author, year^[[1]](#footnote-1)^ | Country | Sample | Timeframe | | Impact on labour Market Outcomes | | | | Moderators | Risk of bias judgment |
|  |  |  | Before COVID-19 pandemic | During COVID-19 pandemic | Employment status | Income | Working hours/ WFH^[[2]](#footnote-2)^ | Variation between groups of individuals | |  |
| Jetha, 2021^A1^ | Canada | Young adults with rheumatic disease (N=133) | December 2019 | March 2020 | Employment decreased from 86.0% to 71.0% | Not reported (N/R) | N\R | N\R | | Serious due to selection |
| Koebel, 2020 ^A2^ | Canada | Statistics Canada’s Labour Force Survey | February and April 2019 | February and April 2020 | N/R | N\R | Working hours declined by 5.1 hours for low earners and by 1.6 hours for high earners | Low earners experienced larger reduction in hours worked compared to high earners | | Moderate |
| Lemieux, 2020 ^A3^ | Canada | Labour Force Survey | February 2020 | April 2020 | Employment decreased by 15.0% | N\R | Weekly working hours declined by 32.0% | lowest earners experienced the greatest job and hour losses | | Moderate |
| Collins, 2020 ^A4^ | US | CPS (N= 2432) | February 2020 | April 2020 | N\R | N\R | Working hours declined for mothers with young children | Mothers with young children have reduced their work hours 4 to 5 times more than fathers | | Moderate |
| Cho et al., 2021 ^A5^ | US | CPS | January 2019 | October 2020 | Employment decreased by 9.6 ppts^[[3]](#footnote-3)^ in non- metropolitan areas and by 14.6 ppts in metropolitan areas | N\R | N\R | Metropolitan areas experienced larger reduction in employment compared to non- metropolitan areas | | Moderate |
| Cowan and Garcia, 2021 ^A6^ | US | CESifo Forum | February (2019-2021) | April (2019-2020) | Likelihood of employment declined by 3.5 ppts for younger people | N\R | N\R | Young people (21–30-year-olds) showed smaller likelihood of employment compared to older people (31–40-year-olds) | | Moderate |
| Delaney, 2021 ^A7^ | US | Faculty, staff, and trainees at University of Utah (N= 5030) | Retrospective data | August, 2020 | 21.0% of respondents moderately or very seriously considered leaving the workforce | N\R | 30.0% of respondents considered reducing working hours | N\R | | Serious due to selection |
| Ge, 2020 ^A8^ | US | U.S. Department of Labor’s and SafeGraph | January 2020 | April 2020 | Unemployment increased | N\R | Part-time work became negatively correlated with WFH capacity | Males, black, non-college-educated, younger, and immigrant workers are more likely to work in occupations most difficult to WFH | | Low |
| Hardy et al., 2021 ^A9^ | US | CPS | January 2019 | April 2021 | Unemployment increased | N\R | N\R | Blacks and Hispanics experienced higher unemployment relative to whites by 2 to 4 ppts | | Moderate |
| Heggeness, 2020 ^A10^ | US | Monthly panel data from CPS | January to May 2019 | January to May 2020 | N\R | N\R | Weekly working hours declined for both mothers and fathers | Mothers in areas with early school closures were 68.8% more likely to have a job but not be working | | Serious due to missing data |
| Hu, 2021 ^A11^ | US | Physicians from CPS (N= 2563) | January 2019 to March 2020 | April to December 2020 | The percentage of physicians reporting being laid off increased from 0.05% to 0.45% | N\R | Working hours declined by 6.0% from January 2019 | N\R | | Moderate |
| Kim, 2021 ^A12^ | US | CPS | January 2020 | August 2020 | Unemployment increased | N\R | N\R | Asian Americans are more negatively affected than any other racial group | | Moderate |
| Lee et al., 2021 ^A13^ | US | CPS (N=80,000) | January 2020 | December 2020 | Unequal Employment Effects | N\R | N\R | Women, minorities, the less educated, younger workers and leisure/hospitality sectors were most affected | | Moderate |
| Lin, 2021 ^A14^ | US | CPS (N=391,915 individual month) | March to June 2016 | March to June 2020 | Unemployment increased | N\R | N\R | Workers in the smallest enterprises experienced an increase of 5.7% in excess unemployment rate | | Moderate |
| Milovanska-Farrington , 2021 ^A15^ | US | CPS (N=>3 million) | January 2018 | June 2020 | Unequal Employment Effects | No change in earnings gaps between ethnic groups | N\R | Widening employment gap between minorities and Whites and those with and without children | | Moderate |
| Costoya, 2021 ^A16^ | Argentina | Couples (N=961) | Retrospective data | June to July 2020 | N\R | N\R | Weekly working hours declined by 7.5 hour for men and by 5.8 hour for women | Larger reduction in hours worked by men than by women | | Moderate |
| Gil et al., 2021^A17^ | Chile | Longitudinal panel study of households living in Chile’s informal settlements (n= 5,622 heads of household) | May 2020 | September 2020 | Employment decreased | N\R | N\R | Larger effects among immigrants, which corresponds to 40% of the sample, and women may face additional challenges | | Moderate |
| Caruso et al., 2021^A18^ | Venezuela | Encuesta Nacional de Condiciones de Vida (ENCOVI) | Retrospective data | 2020 | Employment decreased by 16.5 ppts | N\R | N\R | Self-employed and informally employed were most affected | | Moderate |
| Hoehn-Velasco et al., 2021^A19^ | Mexico | All formal private sector employees in Mexico (administrative data) | February 2020 | November 2020 | Employment decreased by 5.4% | N\R | N\R | The most affected were 15–29- year-olds, >60 years old, low-income earners, workers in certain sectors (e.g., construction, hospitality) | | Low |
| Qian, 2021^A20^ | UK and US | Couples from UKHLS (N=2186) and CPS (N=2,718) | January and February2020 | April and May 2020 | Employment decreased | N\R | N\R | Stronger impact on UK than in the US | | Moderate |
| Adams-Prassl et al., 2020^A21^ | UK | UK workers, wave 1(N=4932), wave 2 (N=4,009) | February 2020 | May 2020 | 35.0% of those in work in February report being furloughed from their main job | N\R | Weekly working hours declined by 44.0% | Women, younger workers, and workers with alternative work arrangements have been more likely to be put on furlough | | Moderate |
| Crossley, 2021^A22^ | UK | UKHLS- COVID-19 study (N= 7,404) | Retrospective data | April and May 2020 | N\R | 50.0% experienced declines in household earnings | N\R | Income declines are most severe in the bottom pre-pandemic income quintiles | | Moderate |
| Hupkau, 2020^A23^ | UK | UKHLS- COVID-19 study (N=2,936) | January 2020 | April to May 2020 | 4.3 % reported being out of work by May 2020 | Drop in earnings by 9.5 % on average (or £36) | Weekly working hours declined by 11.2 hours | N\R | | Serious due to missing data |
| Hupkau, 2020^A24^ | UK | UKHLS- COVID-19 study (N= 8,073) | Retrospective data | April 2020 | N\R | Decline in earnings | N\R | Low educated parents, those on precarious job contracts are more likely to have partial drop in earnings | | Serious due to selection and missing data |
| Jones, 2022^A25^ | UK | Labour Force Survey (N=20,000) | January 2019 | December 2020 | Unequal Employment Effects | N\R | N\R | Disabled workers were more likely to be temporarily away from work | | Low |
| Apouey, 2020^A26^ | France | Gig workers (N=137) | March 2020 | April 2020 | 56.0% had stopped working | Decline in earnings by 28.0% on average | N\R | Bikers were 38.0% more likely to be working outside relative to a mean of 10.0% for other workers | | Low |
| Bonacini, 2021^A27^ | Italy | Participation, Labour and Unemployment Survey and Labour Force Survey (N=14,307) | February 2020 | May 2020 | N\R | High WFH feasibility is associated with an increase in average labour income | High WFH feasibility in more COVID-19-infected areas | Employees in more COVID-19-infected areas report higher WFH feasibility and labour income on average than the ones living in less-affected areas | | Moderate |
| Del Boca, 2020^A28^ | Italy | Italian coupled women (N= 800) | April to July 2019 | April 2020 | Unequal Employment Effects | N\R | Unequal effects | 44.0% of working women have kept their jobs by working from home (vs. 30.0% of men) | | Serious due to selection |
| Bauer and Weber, 2021^A29^ | Germany | The Establishment History Panel (BHP) | March 2020 | April 2020 | Unemployment inflow increased by 60.0% | N\R | N\R | N\R | | Moderate |
| Graeber et al., 2021^A30^ | Germany | The Socio-Economic Panel (SOEP)-CoV survey | April 2020 | July 2020 | N\R | Unequal effects | Unequal effects | Self-employed individuals are 42 ppts more likely to have experienced an income loss and 30 ppts more likely to have experienced a reduction in working hours compared to employees | | Moderate |
| Hassink, 2020^A31^ | Netherlands | Statistics Netherlands | January to March 2019 | January to March 2020 | Employment decreased by 2 ppts | Hourly wages declined by 0.25 euro | Monthly working hours declined by 1.75 hours | N\R | | Moderate |
| Zimpelmann et al., 2021^A32^ | Netherlands | Longitudinal Internet Studies for the Social Sciences (LISS) panel dataset (N=7,000) | January 2020 | December 2020 | N\R | N\R | Working hours were more substantially affected than employment | People with lower socioeconomic status were most affected in terms of working hours | | Moderate |
| Clark et al., 2021^A33^ | France, Germany, Italy and Spain | COME-HERE (COVID-19, Mental Health, Resilience and self-regulation) panel survey | January 2020 | January 2021 | Unequal employment effects | N\R | N\R | Self-employed were hit the hardest. Italy was most affected | | Moderate |
| Su, 2021^A34^ | France, Germany, Italy, Spain, and UK | OECD database and the European Centre for Disease Prevention and Control database | December 2019 | December 2020 | Unemployment increased | N\R | N\R | Larger impact in Germany (1.1%), Spain (1.4%), and UK (0.49%) compared to France and Italy | | Moderate |
| Aldan et al., 2021^A35^ | Turkey | Working-age population (15+) from household Labour Force Survey | Retrospective data | 2nd, 3rd and 4th quarters of 2020 | Unemployment increased by 0.6 ppts | N\R | N\R | Women, young workers and less educated were most affected | | Moderate |
| Janssens, 2021^A36^ | Kenya | Financial and Health Diaries (N= 2,995) | 6 weeks before 1^st^ case | 5 weeks after lockdown | N\R | Income from work decreased with almost one-third | N\R | N\R | | Serious due to missing data |
| Balde, 2020^A37^ | Senegal, Mali, and Burkina Faso | Innovative online survey (N=900) | Retrospective data | April to May 2020 | 25.0% lost their jobs | 55.0% experienced decrease in earning | N\R | Job loss is higher in Senegal (29.0%), then by Mali (23.0%) and Burkina Faso (22.0%) | | Serious due to selection |
| Posel, 2021^A38^ | South Africa | National Income Dynamics Study (NIDS) (N= 2213) | Retrospective data | May to August 2020 | 30.0% lost their jobs, and 12.0% were furloughed | N\R | N\R | N\R | | Serious due to selection |
| Barker, 2020^A39^ | Bangladesh and Nepal | Three household databases were combined (two samples in Bangladesh and one in Nepal) | Retrospective data | April to May 2020 | N\R | Decline in income | N\R | Bangladesh sample showed 36.0% decline in monthly income, Nepal sample showed 25.0% decline in earnings among migrant households. | | Moderate |
| Hamadani, 2020^A40^ | Bangladesh | Mothers of children in a randomised controlled trial (N= 2424) | Retrospective data | May to June 2020 | N\R | 96.0% reported reduction in the family paid work | N\R | N\R | | Moderate |
| Habibullah, 2021^A41^ | Malaysia | Employment Insurance System (N= 230) | January 2020 | September 2020 | Unemployment increased from 0.35% to 1.1% for every 1.0% increase in the lockdown measures | N\R | N\R | N\R | | Moderate |
| He, 2020^A42^ | China | Domestic helpers (N= 46,288) | November 2019 | June 2020 | Decline in pattern of short-term labour supply | Decline in income by 1.6% | N\R | N\R | | Moderate |
| Dang, 2021^A43^ | China, South Korea, Japan, Italy, UK, US | Selected samples from each country (N=6089) | Retrospective data | April 2020 | Unequal employment effects | Decline in income | N\R | Women are 24.0% more likely to permanently lose their job and expected their labour income to fall by 50.0% more than men do | | Moderate |
| Fukai et al., 2021^A44^ | Japan | Labour Force Survey | Retrospective data | June 2020 | Employment probability declined by 10.0% | N\R | N\R | Younger and female respondents are more affected than are older and male respondents | | Moderate |
| Hoshi et al. 2022^A45^ | Japan | Labour Force Survey (N=40,000 households) | February 2019 | June 2020 | Change in employment not statistically significant | N\R | Working hours declined | Greater decrease in working hours for executives, owners, and 31–45-year-olds; rise in unemployment greatest among >60-year-olds and people in service and sales occupations | | Low |
| Lee and Yang, 2022^A46^ | South Korea | Monthly Economically Active Population Survey (EAPS) (N=35,000 households) | April 2019 | December 2020 | Employment loss of 3.2% of the entire number of employed persons | N\R | Working hours declined by 2.3 -5.7 hours | Temporary workers, women and less educated workers were most affected. | | Low |
| Guven, 2020^A47^ | Australia | Labour Force Survey (N= 279368) | January 2019 to March 2020 | March to May 2020 | Unemployment increased by 1.1% | N\R | Weekly working hours declined by 1-hour | Unemployment is larger for immigrants and individuals with occupations unsuitable for remote work | | Moderate |
| Wolfe, 2021^A48^ | Global | UKHLS-COVID19 survey(N=4806), individuals from the other five countries (N=1794). | Retrospective data | April to May 2020 | N\R | Unequal effects | N\R | Self-employed are more likely to report higher expected fall in own income | | Moderate |

| Table A2 Summary of descriptive studies’ characteristics | | | | | | | | | | |
| --- | --- | --- | --- | --- | --- | --- | --- | --- | --- | --- |
| First author, year^[[4]](#footnote-4)^ | Country | Sample | Timeframe | | Impact on labour Market Outcomes | | | | Moderators | Risk of bias overall judgment |
|  |  |  | Before COVID-19 pandemic | During COVID-19 pandemic | Employment | Income | Working hours/ WFH^[[5]](#footnote-5)^ | Variation between groups of individuals | |  |
| Abrams, 2021^A49^ | US | COVID-19 Coping Study (N=6,264) | Retrospective data | April and May 2020 | 5.9% lost their job, 18.3% were furloughed or placed on a leave of absence | 22.8% reported reductions in income | 22.8% experienced reductions in their hours and 27.1% transitioned to WFH | Job loss and reduced hours/income were more common among Hispanics compared to other racial/ethnic groups | | Serious due to selection and missing data |
| Dias, 2021^A50^ | US | CPS^[[6]](#footnote-6)^ | January 2020 | August 2020 | Unequal employment effects | N\R | N\R | Black, Hispanic, and Asian workers are more likely to be laid off compared with Whites | | Low |
| Bhandari,2021^A51^ | US | Current Population Survey (CPS) (N= 418,899) | July 2019 | July 2020 | Unemployment rates increased for all healthcare industries | N\R | N\R | Dentists’ offices (41.3%), outpatient centres (10.5%), physician offices (9.5%), and home health (7.8%) | | Low |
| Bell, 2020^A52^ | US and UK | Online poll | Retrospective data | April 2020 | Unequal employment effects | Unequal effects | N\R | Third of US workers report losing at least half of their income, compared with a quarter in the UK | | Low |
| Borjas, 2020^A53^ | US | CPS Basic Monthly Files | January 2020 | April 2020 | Employment rate for immigrant men decreased from 88.6% to 85.3% | N\R | N\R | The immigrant-native employment gap fell by 7.9 ppts | | Moderate |
| Bui, 2020^A54^ | US | Older workers from CPS | Retrospective data | April 2020 | Unemployment rates of 16.8% for women and 14.2% for men | N\R | N\R | Older workers, especially women, were more impacted | | Low |
| Gemelas, 2021^A55^ | US | The Bureau of Labor Statistics CPS (N= 60,000) | January to March 2020 | April to June 2020 | The number of employed workers decreased by 12.0% | N\R | N\R | Black or African American, Asian American, and Hispanic were more impacted | | Moderate |
| Groshen, 2020^A56^ | US | Bureau of Labor Statistics’ (N=60,000) | Retrospective data | September 2020 | Unemployment rate increased from 3.5% to 14.7%. | N\R | N\R | Hispanic, African American and women workers’ jobs were more impacted | | Moderate |
| Moen, 2020^A57^ | US | CPS (N= 175,533) | January 2020 | April 2020 | Unequal employment effects | N\R | N\R | Black men aged 20–29 with a college degree experienced a 12.4 % increase in being not in the labour force | | Moderate |
| Shockley, 2021^A58^ | US | Couples who have at least one child under age 6 years (N=274 couple) | March 2020 | May 2020 | N\R | N\R | Reduction in working hours for both partners | N\R | | Moderate |
| Winkelmann and Games, 2020^A59^ | US | Athletic Trainers (N=611) | Retrospective data | April 2020 | 20.0% were unable to work at all | N\R | N\R | 56.6% were in secondary schools, 18.0% were in colleges or universities, and 11.5% were in clinics | | Moderate |
| Gomes, 2020^A60^ | Brazil | Brazilian Society of Urology (N=766) | Retrospective data | April 2020 | Reduction ≥ 50.0% of patient visits | 50.0% reduction in income was reported by 54.3% urologists | N\R | Urologists from the highest COVID-19 incidence states were at a higher risk to have a reduction of patient visits | | Moderate |
| Romero, 2021^A61^ | Brazil | ConVid – Behavior Survey (N= 9173) | Retrospective data | April to May 2020 | N\R | 23.5% reduction in household income for half of older adults | 21.2% worked from home | The percentage of those formally employed was higher among men | | Moderate |
| Bottan, 2020^A62^ | 17 countries in Latin America and the Caribbean | Online survey (N= 230,540). | Retrospective data | March to April, 2020 | 45.0% of respondents report that a at least one household member lost a job | N\R | N\R | Among households owning small businesses ,59.0% of respondents reported that a household member closed their business | | Serious due to selection |
| Spiro, 2021^A63^ | UK | HEartS Professional survey (N= 385) | Retrospective data | April to June 2020 | N\R | 76.0% had experienced a decrease in income | 71.0% spent less time working than before | N\R | | Moderate |
| Gray et al., 2021^A64^ | Wales | Nationally representative household survey (n=1379) | May 2020 | June 2020 | 3.3% experienced unemployment and 24.0% reported being placed on furlough | N\R | N\R | N\R | | Moderate |
| Aspachs, 2021^A65^ | Spain | Bank account data from CaixaBank (N=3 million) | February 2020 | June 2020 | N\R | Income inequality increases by 25.0% | N\R | Inequality increases much more among young, workers and foreign born than among natives and in regions that rely heavily on tourism | | Serious due to selection and classification |
| Dolado, 2020^A66^ | Spain | Labour Force Survey | The 4^th^ quarter of 2019 | The 2^nd^ quarter of 2020 | Unemployment rate increased by 2.5 ppts | N\R | N\R | N\R | | Moderate |
| Palomino, 2021^A67^ | Spain | The European survey of Income and Living Conditions | Retrospective data | 2 months lockdown+ 10 months after lockdown | N\R | 10.6 ppts increase in workers with incomes below the national poverty line | N\R | Inequality between regions increased | | Moderate |
| Faber, 2020^A68^ | Switzerland | Swiss Labour Force Survey | Retrospective data | April 2020 | 31.0% of jobs in Switzerland have been potentially restricted | N\R | N\R | Strongest effects for the large industries hospitality, construction, and arts and entertainment. | | Moderate |
| Novikova, 2021^A69^ | Ukraine and the EU countries | European System of Social Indicators | Retrospective data | The 2^nd^ quarter of 2020 | Unequal employment effects | N\R | Unequal effects | Ukraine showed higher risk of unemployment (35.1%) and loss of working time (25.0%) compared to the EU countries | | Moderate |
| Ranchhod, 2021^A70^ | South Africa | NIDS-CRAM study (N= 6,000) | February 2020 | March to April 2020 | Employment decreased from 57.0% in February to 48.0% in April 2020 | N\R | N\R | Women, African/Blacks, youth, and less educated groups more impacted | | Moderate |
| Anand and Thampi, 2021^A71^ | India | National Sample Survey Ofce (NSSO) | Retrospective data | 2020-2021 | N\R | Unequal effects | N\R | Women earnings were 63.0% of men earnings and rural earnings were only half of urban earnings in 2018–2019 | | Moderate |
| Behera et al., 2021^A72^ | India | National Sample Survey Ofce (NSSO) | Retrospective data | 2019-2020 | Employment decreased by 0.34%, with a loss of 1.56 million jobs | N\R | N\R | N\R | | Moderate |
| Chandra et al.,2021^A73^ | India | Patients diagnosed with COVID-19 (n=190) | May 2020 | July 2020 | Six patients (3.2%) lost their job permanently | 30.5% of patients reported deduction in their salary | N\R | N\R | | Moderate |
| Deshpande, 2020^A74^ | India | Consumer Pyramids Household Survey (N=43,000) | Retrospective data | March 2020 | Unequal employment effects | N\R | N\R | Women who were employed in the pre-lockdown phase were roughly 20.0% less likely to be employed in the post-lockdown phase | | Low |
| Mamgain, 2021^A75^ | India | Consumer Pyramids Household Survey (N= 174,405) | April to December 2019 | March to June 2020 | 31.0% of lost employment | N\R | N\R | Small traders, self-employed, migrant workers, daily wage labourers, youth, and women more impacted | | Serious due to selection bias |
| Srivastava, 2021^A76^ | India | Domestic workers (N=75) | Retrospective data | December 2020 | 31.0% of respondents had not found any work | No alternate means of earning income. | N\R | 74.0% were not called back were involved in cooking food due to fears of high virus transmission | | Moderate |
| Kikuchi, 2021^A77^ | Japan | Labour Force Survey and Employment Status Survey | Retrospective data | The first quarter of 2020 | Regular workers’ employment declined by around 1.0% in April and May compared to January | In April 2020, the average earnings for all sectors declined by 4.0% compared to the April 2019 | N\R | Female, contingent workers and low-skilled workers were the most impacted | | Moderate |
| Tsurugano, 2021^A78^ | Japan | Labour Force Survey – Working students (N= 2530) | January 2019 | May 2020 | Significant reductions in the numbers of young working students (−49.0%) | N\R | N\R | N\R | | Moderate |
| Borland, 2020^A79^ | Australia | Labour Force Survey | Retrospective data | March to June 2020 | Employment/population rate of young workers decreased by 9.8 ppts | N\R | N\R | Young people, females and workers in occupations such as community and personal services and sales are most affected | | Low |
| Childs et al., 2021^A80^ | Australia | Australasian Sonographers (n=444) | March 2020 | June 2020 | N\R | N\R | Working hours decreased for 68.0% of the sonographers | N\R | | Moderate |
| Churchill, 2020^A81^ | Australia | Labour Force data from the Australian Bureau of Statistics | February 2020 | June 2020 | The labour force participation rates decreased | N\R | N\R | The unemployment of younger Australians was significantly more impacted than older age groups | | Low |
| Alon et al., 2021^A82^ | Global | Micro survey data from 28 countries | 4^th^ quarter of 2019 | 2^nd^ quarter of 2020 | Employment decreased by more than 5.0% in the US, Canada, and Spain and by less than 1.0% in Germany and the UK | N\R | Working hours declined by 36.0% in the US and more than 50.0% in Germany | Women’s labour supply fell relative to men’s in 18 of 28 countries when measured by employment, and in 19 of 28 countries when measured by hours worked. | | Moderate |
| Cohen, 2021^A83^ | Global | International Labour Organisation | April 2019 | April 2020 | Unequal employment effects | N\R | N\R | Women experienced an average 5.0% reduction in employment compared to 3.9% for men | | Low |
| Ogando et al., 2022^A84^ | Global | Telephone survey respondents from lists of membership-based organisations of informal workers (N=2,292) | January 2020 | July 2020 | N\R | Reduced earnings during the peak of COVID-19 restrictions | Fewer working days during the peak of COVID-19 restrictions | Women were most affected | | Serious due to selection bias |
| Ud Din, 2020^A85^ | Global | ILO and World Bank data | Retrospective data | March 2020 | Unequal employment effects | N\R | N\R | Lower-middle-income, middle income and upper-middle- income group countries more impacted | | Moderate |

**List of references of included papers in table A1 and A2**

**A1.** Jetha A, Tucker L, Chen C, Gignac M. Impact of the COVID‐19 Pandemic on the Employment of Canadian Young Adults with Rheumatic Disease: Findings from a Longitudinal Survey. *Arthritis Care & Research* 2021;73(8):1146-1152.

**A2.** Koebel K, Pohler D. Labor Markets in Crisis: The Double Liability of Low‐Wage Work During COVID‐19. *Industrial Relations: A Journal of Economy and Society* 2020;59(4):503-531.

**A3.** Lemieux T, Milligan K, Schirle T, Skuterud M. Initial Impacts of the COVID-19 Pandemic on the Canadian Labour Market. *Canadian Public Policy* 2020;46(s1): S55-S65.

**A4.** Collins C, Landivar L, Ruppanner L, Scarborough W. COVID‐19 and the gender gap in work hours. *Gender, Work & Organization* 2020;28(S1):101-112.

**A5.** Cho SJ, Lee JY, Winters JV. Employment impacts of the COVID-19 pandemic across Metropolitan status and size. *SSRN Electronic Journal* 2020;

**A6.** Cowan B, Garcia KS. How Has Covid-19 Affected Young Workers? *CESifo Forum* 2022.

**A7.** Delaney R, Locke A, Pershing M, Geist C, Clouse E, Precourt Debbink M et al. Experiences of a Health System’s Faculty, Staff, and Trainees’ Career Development, Work Culture, and Childcare Needs During the COVID-19 Pandemic. *JAMA Network Open* 2021;4(4):e213997.

**A.8** Ge S, Zhou Y. Social Distancing, Labor Market Outcomes, and Job Characteristics in the COVID-19 Pandemic. *SSRN Electronic Journal* 2020.

**A9.** Hardy B, Hokayem C, Roll S. Crashing without a parachute: Racial and educational disparities in unemployment during COVID-19*. The ANNALS of the American Academy of Political and Social Science* 2021;698(1):39–67.

**A10.** Heggeness M. Estimating the immediate impact of the COVID-19 shock on parental attachment to the labor market and the double bind of mothers. *Review of Economics of the Household* 2020;18(4):1053-1078.

**A11.** Hu X, Dill M. Changes in Physician Work Hours and Patterns During the COVID-19 Pandemic. *JAMA Network Open* 2021;4(6):e2114386.

**A12.** Kim A, Kim C, Tuttle S, Zhang Y. COVID-19 and the decline in Asian American employment. *Research in Social Stratification and Mobility* 2021;71:100563.

**A13.** Lee SY, Park M, Shin Y. Hit harder, recover slower? unequal employment effects of the covid-19 shock. *Federal Reserve Bank of St. Louis REVIEW* 2021.

**A14.** Lin K, Aragão C, Dominguez G. Firm Size and Employment during the Pandemic. *Socius: Sociological Research for a Dynamic World* 2021;7:237802312199260.

**A15.** Milovanska-Farrington S. The effect of covid-19 as an economic shock on the gender and ethnic gap in labour market outcomes. *Studies in Microeconomics* 2021;9(2):227–55.

**A16.** Costoya V, Echeverría L, Edo M, Rocha A, Thailinger A. Gender Gaps within Couples: Evidence of Time Re-allocations during COVID-19 in Argentina. *Journal of Family and Economic Issues* 2021.

**A17.** Gil Mc Cawley D, Dominguez P, Undurraga EA, Valenzuela E. Employment loss in informal settlements during the covid-19 pandemic: Evidence from Chile. *SSRN Electronic Journal*. 2021.

**A18.** Caruso G, Chittaro L, Cucagna ME, Espana LP. From bad to worse: The economic impact of covid-19 in developing countries. evidence from Venezuela. *Latin American Economic Review* 2021;1–22.

**A19.** Hoehn-Velasco L, Silverio-Murillo A, Balmori de la Miyar JR. The long downturn: The impact of the great lockdown on formal employment. *Journal of Economics and Business* 2021; 115:105983.

**A20.** Qian Y, Hu Y. Couples' changing work patterns in the United Kingdom and the United States during the COVID‐19 pandemic. *Gender, Work & Organization* 2021;28(S2):535-553.

**A21.** Adams‐Prassl A, Boneva T, Golin M, Rauh C. Furloughing*. *Fiscal Studies* 2020;41(3):591-622.

**A22.** Crossley T, Fisher P, Low H. The Heterogeneous and Regressive Consequences of COVID-19: Evidence from High Quality Panel Data. *SSRN Electronic Journal* 2020.

**A23.** Hupkau C, Isphording I, Machin S, Ruiz-Valenzuela J. Labour Market Shocks During the Covid-19 Pandemic, Inequalities and Child Outcomes. *SSRN Electronic Journal*. 2021.

**A24.** Hupkau C, Petrongolo B. Work, Care and Gender During the Covid-19 Crisis. *SSRN Electronic Journal*. 2020.

**A25.** Jones M. Covid-19 and the labour market outcomes of disabled people in the UK. *Social Science & Medicine*. 2022; 292:114637.

**A26.** Apouey B, Roulet A, Solal I, Stabile M. Gig Workers During the COVID-19 Crisis in France: Financial Precarity and Mental Well-Being. SSRN Electronic Journal. 2020.

**A27.** Bonacini L, Gallo G, Scicchitano S. Working from home and income inequality: risks of a ‘new normal’ with COVID-19. *Journal of Population Economics* 2020;34(1):303-360.

**A28.** Del Boca D, Oggero N, Profeta P, Rossi M. Women’s and men’s work, housework and childcare, before and during COVID-19. *Review of Economics of the Household* 2020;18(4):1001-1017.

**A29.** Bauer A, Weber E. Covid-19: How much unemployment was caused by the shutdown in Germany? *Applied Economics Letters* 2020;28(12):1053–8.

**A30.** Graeber D, Kritikos AS, Seebauer J. Covid-19: A crisis of the female self-employed. *SSRN Electronic Journal* 2020.

**A31.** Hassink W, Kalb G, Meekes J. The Dutch Labour Market Early on in the COVID-19 Outbreak: Regional Coronavirus Hotspots and the National Lockdown. *SSRN Electronic Journal*. 2020.

**A32.** Zimpelmann C, Gaudecker H-Mvon, Holler R, Janys L, Siflinger B. Hours and income dynamics during the COVID-19 pandemic: The case of the netherlands. *Labour Economics* 2021; 73:102055.

**A33.** Clark AE, D’Ambrosio C, Lepinteur A. The fall in income inequality during COVID-19 in four European countries. *The Journal of Economic Inequality* 2021;19(3):489–507.

**A34.** Su C, Dai K, Ullah S, Andlib Z. COVID-19 pandemic and unemployment dynamics in European economies. *Economic Research-Ekonomska Istraživanja* 2021;1-13.

**A35.** Aldan A, Çıraklı ME, Torun H. Covid 19 and the Turkish labor market: Heterogeneous effects across demographic groups. *Central Bank Review* 2021;21(4):155–63.

**A36.** Janssens W, Pradhan M, de Groot R, Sidze E, Donfouet H, Abajobir A. The short-term economic effects of COVID-19 on low-income households in rural Kenya: An analysis using weekly financial household data. *World Development* 2021;138:105280.

**A37.** Balde R, Mohamed B, Elvis A. Labour market effects of COVID‐19 in sub‐Saharan Africa: An informality lens from Burkina Faso, Mali and Senegal. *UNU-MERIT Working Papers* 2020.

**A38.** Posel D, Oyenubi A, Kollamparambil U. Job loss and mental health during the COVID-19 lockdown: Evidence from South Africa. *PLOS ONE 2021*;16(3): e0249352.

**A39.** Barker N, Davis C, López-Peña P, Mitchell H, Mobarak A, Mushfiq N et al. Migration and the labour market impacts of covid-19. *WIDER Working Paper No 2020/139* 2020.

**A40.** Hamadani J, Hasan M, Baldi A, Hossain S, Shiraji S, Bhuiyan M et al. Immediate impact of stay-at-home orders to control COVID-19 transmission on socioeconomic conditions, food insecurity, mental health, and intimate partner violence in Bangladeshi women and their families: an interrupted time series. *The Lancet Global Health* 2020;8(11):e1380-e1389.

**A41.** Habibullah M, Saari M, Din B, Safuan S, Utit C. Labour Market Reactions to Lockdown Measures during the Covid-19 Pandemic in Malaysia: An Empirical Note. *Journal Ekonomi Malaysia* 2021;55(1):1-12.

**A42.** He X, Xiao F. Unintended Consequences of Lockdowns: Evidence from Domestic Helpers in Urban China. *Frontiers of Economics in China* 2020;15(4).

**A43.** Dang H, Nguyen C. Gender Inequality During the Covid-19 Pandemic: Income, Expenditure, Savings, and Job Loss. *SSRN Electronic Journal*. 2020.

**A44.** Fukai T, Ichimura H, Kawata K. Describing the impacts of COVID-19 on the labor market in Japan until June 2020. *The Japanese Economic Review* 2021;72(3):439–70.

**A45.** Hoshi K, Kasahara H, Makioka R, Suzuki M, Tanaka S. The heterogeneous effects of COVID-19 on Labor Markets: People’s Movement and non-pharmaceutical interventions. *Journal of the Japanese and International Economies* 2022;63:101170.

**A46.** Lee J, Yang H-S. Pandemic and employment: Evidence from covid-19 in South Korea. *Journal of Asian Economics* 2022;78:101432.

**A47.** Guven C, Sotirakopoulos P, Ulker A. Short-term Labour Market Effects of COVID-19 and the Associated National Lockdown in Australia: Evidence from Longitudinal Labour Force Survey. *GLO Discussion Paper, No 635* 2020.

**A48.** Wolfe M, Patel P. Everybody hurts: Self-employment, financial concerns, mental distress, and well-being during COVID-19. *Journal of Business Venturing Insights* 2021;15:e00231.

**A49.** Abrams L, Finlay J, Kobayashi L. Job Transitions and Mental Health Outcomes Among U.S. Adults Aged 55 and Older During the COVID-19 Pandemic. *The Journals of Gerontology: Series B* 2021.

**A50.** Dias F. The Racial Gap in Employment and Layoffs during COVID-19 in the United States: A Visualization. *Socius: Sociological Research for a Dynamic World* 2021;7:237802312098839.

**A51.** Bhandari N, Batra K, Upadhyay S, Cochran C. Impact of COVID-19 on Healthcare Labor Market in the United States: Lower Paid Workers Experienced Higher Vulnerability and Slower Recovery. *International Journal of Environmental Research and Public Health* 2021;18(8):3894.

**A52.** Bell D, Blanchflower D. US AND UK LABOUR MARKETS BEFORE AND DURING THE COVID-19 CRASH. *National Institute Economic Review* 2020;252: R52-R69.

**A53.** Borjas G, Cassidy H. The Adverse Effect of the COVID-19 Labor Market Shock on Immigrant Employment. *SSRN Electronic Journal* 2020.

**A54.** Bui T, Button P, Picciotti E. Early Evidence on the Impact of Coronavirus Disease 2019 (COVID-19) and the Recession on Older Workers. *Public Policy & Aging Report* 2020;30(4):154-159.

**A55.** Gemelas J, Davison J, Keltner C, Ing S. Inequities in Employment by Race, Ethnicity, and Sector During COVID-19. *Journal of Racial and Ethnic Health Disparities* 2021;9(1):350-355.

**A56.** Groshen E. COVID-19’s impact on the U.S. labor market as of September 2020. *Business Economics* 2020;55(4):213-228.

**A57.** Moen P, Pedtke J, Flood S. Disparate Disruptions: Intersectional COVID-19 Employment Effects by Age, Gender, Education, and Race/Ethnicity. *Work, Aging and Retirement* 2020;6(4):207-228.

**A58.** Shockley K, Clark M, Dodd H, King E. Work-family strategies during COVID-19: Examining gender dynamics among dual-earner couples with young children. *Journal of Applied Psychology* 2021;106(1):15-28.

**A59.** AWinkelmann Z, Games K. Athletic Trainers' Job Tasks and Status During the COVID-19 Pandemic: A Preliminary Analysis. *Journal of Athletic Training* 2020;56(1):20-30.

**A60.** Gomes C, Favorito L, Henriques J, Canalini A, Anzolch K, Fernandes R et al. Impact of COVID-19 on clinical practice, income, health and lifestyle behavior of Brazilian urologists. *International braz j urol* 2020;46(6):1042-1071.

**A61.** Romero D, Muzy J, Damacena G, de Souza N, de Almeida W, Szwarcwald C et al. Older adults in the context of the COVID-19 pandemic in Brazil: effects on health, income and work. *Cadernos de Saude Publica* 2021;37(3).

**A62.** Bottan N, Hoffmann B, Vera-Cossio D. The unequal impact of the coronavirus pandemic: Evidence from seventeen developing countries. *PLOS ONE* 2020;15(10):e0239797.

**A63.**Spiro N, Perkins R, Kaye S, Tymoszuk U, Mason-Bertrand A, Cossette I et al. The Effects of COVID-19 Lockdown 1.0 on Working Patterns, Income, and Wellbeing Among Performing Arts Professionals in the United Kingdom (April–June 2020). *Frontiers in Psychology* 2021;11.

**A64.** Gray BJ, Kyle RG, Song J, Davies AR. Characteristics of those most vulnerable to employment changes during the COVID-19 pandemic: A Nationally Representative cross-sectional study in Wales. *J Epidemiol Community Health* 2022; 76:8–15.

**A65.** Aspachs O, Durante R, Graziano A, Mestres J, Reynal-Querol M, Montalvo J. Tracking the impact of COVID-19 on economic inequality at high frequency. *PLOS ONE* 2021;16(3):e0249121.

**A66.** Dolado J, Felgueroso F, Jimeno J. Past, present and future of the Spanish labour market: when the pandemic meets the megatrends*. Applied Economic Analysis*. 2021; 29 (85): 21-41

**A67.** Palomino J, Rodriguez J, Sebastian R. The COVID-19 Shock on the Labour Market: Poverty and Inequality Effects across Spanish Regions. *SSRN Electronic Journal* 2021.

**A68.** Faber M, Ghisletta A, Schmidheiny K. A lockdown index to assess the economic impact of the coronavirus. *Swiss Journal of Economics and Statistics* 2020;156(1).

**A69.** Novikova O, Khandii O, Shamileva L. Comparative Analysis of the Changes of Socioeconomic Risks Caused by the COVID-19 pandemic in Ukraine and the EU Countries. *European Journal of Sustainable Development* 2021;10(2):147-158.

**A70.** Ranchhod V, Daniels R. Labour Market Dynamics in South Africa at the Onset of the COVID‐19 Pandemic. *South African Journal of Economics* 2021;89(1):44-62.

**A71.** Anand I, Thampi A. Pandemic and the crisis of extreme inequality in India. *SSRN Electronic Journal* 2020.

**A72.** Behera DK, Sabreen M, Sharma D. The impact of covid-19 on the Indian economy. *The Political Economy of Covid-19* 2022;302–17.

**A73.** Kumar A, Chandra A, Sarda R, Bir M, Parija PP, Pal A, et al. Impact of covid-19 on the PATIENTSF income and work in Delhi, India. *Journal of Family Medicine and Primary Care* 2021;10(8):3047.

**A74.** Deshpande A. Early Effects of Lockdown in India: Gender Gaps in Job Losses and Domestic Work. *The Indian Journal of Labour Economics* 2020;63(S1):87-90.

**A75.** Mamgain R. Understanding labour market disruptions and job losses amidst COVID-19. *Journal of Social and Economic Development* 2021;23(S2):301-319.

**A76.** Srivastava P, Shukla P. Crisis behind closed doors domestic workers' struggles during the pandemic and beyond. *Economic and Political Weekly* 2021;56(22):17-21.

**A77.** Kikuchi S, Kitao S, Mikoshiba M. Who suffers from the COVID-19 shocks? Labor market heterogeneity and welfare consequences in Japan. *Journal of the Japanese and International Economies* 2021;59:101117.

**A78.** Tsurugano S, Nishikitani M, Inoue M, Yano E. Impact of the COVID‐19 pandemic on working students: Results from the Labour Force Survey and the student lifestyle survey. *Journal of Occupational Health* 2021;63(1).

**A79.** Borland J, Charlton A. The Australian Labour Market and the Early Impact of COVID‐19: An Assessment. *Australian Economic Review* 2020;53(3):297-324.

**A80.** Childs J, Lamb K, Osborne B, Maranna S, Esterman A. The initial impact of covid ‐19 on Australasian Sonographers Part 1: Changes in scan numbers and sonographer work hours. *Sonography*  2021;8(3):90–9.

**A81.** Churchill B. COVID‐19 and the immediate impact on young people and employment in Australia: A gendered analysis. *Gender, Work & Organization* 2020;28(2):783-794.

**A82.** Alon T, Coskun S, Doepke M, Koll D, Tertilt M. From Mancession to Shecession: Women's Employment in Regular and Pandemic Recessions. *SSRN Electronic Journal* 2021.

**A83.** Cohen J, van der Meulen Rodgers Y. The feminist political economy of Covid-19: Capitalism, women, and work. *Global Public Health* 2021;16(8-9):1381-1395.

**A84.** OGANDO AC, ROGAN M, MOUSSIÉ R. Impacts of the Covid‐19 pandemic and unpaid care work on informal workers' livelihoods. *International Labour Review* 2022;161(2):171–94.

**A85.** Ud Din N, Cheng X, Nazneen S, Sun H. COVID-19 Crisis Shifts the Career Paradigm of Women and Maligns the Labour Market: A Gender Lens. *SSRN Electronic Journal* 2020;.

1. ## List of references of included papers numbered from A1 to A85 (see supplementary material - Appendix 3).

   [↑](#footnote-ref-1)
2. Work from home [↑](#footnote-ref-2)
3. ppts=Percentage points [↑](#footnote-ref-3)
4. ## List of references of included papers numbered from A1 to A85 (see below).

   [↑](#footnote-ref-4)
5. Work from home [↑](#footnote-ref-5)
6. Current Population Survey [↑](#footnote-ref-6)
